# Forecasting Short-Term Traffic Flow by Fuzzy Wavelet Neural Network with Parameters Optimized by Biogeography-Based Optimization Algorithm

**DOI:** 10.1155/2018/5469428

**Published:** 2018-10-08

**Authors:** Jeng-Fung Chen, Shih-Kuei Lo, Quang Hung Do

**Affiliations:** ^1^Department of Industrial Engineering and Systems Management, Feng Chia University, Taichung 40724, Taiwan; ^2^Faculty of Information Technology, University of Transport Technology, Hanoi 100000, Vietnam

## Abstract

Forecasting short-term traffic flow is a key task of intelligent transportation systems, which can influence the traveler behaviors and reduce traffic congestion, fuel consumption, and accident risks. This paper proposes a fuzzy wavelet neural network (FWNN) trained by improved biogeography-based optimization (BBO) algorithm for forecasting short-term traffic flow using past traffic data. The original BBO is enhanced by the ring topology and Powell's method to advance the exploration capability and increase the convergence speed. Our presented approach combines the strengths of fuzzy logic, wavelet transform, neural network, and the heuristic algorithm to detect the trends and patterns of transportation data and thus has been successfully applied to transport forecasting. Other different forecasting methods, including ANN-based model, FWNN-based model, and WNN-based model, are also developed to validate the proposed approach. In order to make the comparisons across different methods, the performance evaluation is based on root-mean-squared error (RMSE), mean absolute percentage error (MAPE), and correlation coefficient (*R*). The performance indexes show that the FWNN model achieves lower RMSE and MAPE, as well as higher *R*, indicating that the FWNN model is a better predictor.

## 1. Introduction

In the transportation area, attention is not only paid to construct physical system capacity but also to improve operational efficiency and integration. The intelligent transportation system (ITS) applying the advanced sensing, analysis, control, and communications technologies aims to ease traffic congestion, improve traffic management, and reduce environmental impact. As ITSs have been widely developed throughout the world, how to improve the ability to predict traffic flow in the short term (within the next one hour, e.g., 5 min, 10 min, and 15 min) has been getting much attention from researchers. Short-term traffic flow forecasts can support proactive transportation management and comprehensive traveler information service. The goal is to predict traffic conditions in a transport network based on its past behavior. Several methods have been implemented for the short-term traffic flow forecasts. These methods can be grouped into two categories: (1) methods based on statistical techniques and (2) methods based on artificial intelligence techniques.

With the assumption that the characteristics of forecasting traffic flow data are similar to historical and current flow data, forecasting models based on statistical techniques utilize mathematical statistics to deal with the pervious and current measurements of traffic flow and forecast the future values of traffic flow. Several well-known classical time-series approaches are the Box–Jenkins method [1], autoregressive integrated moving average (ARIMA) model [2, 3], seasonal ARIMA (SARIMA) [4, 5], and a number of variant forms of ARIMA models. Afterwards, researchers found that ARIMA model cannot tackle the problem of forecasting the extreme volume values [6, 7]. Moreover, the short-term traffic flow forecast is more easily affected by the stochastic interferential factors than the long-term one, the uncertainty is greater, and the disciplinarian laws are less obvious. Therefore, using the short-term traffic forecasting models based on the classical mathematical methods such as statistical techniques, the precision of forecast cannot meet the requirement of real-time transportation management systems [8].

As for the use of artificial intelligence-based techniques, several approaches have been applied to the task of traffic forecasting. Artificial neural network (ANN) is certainly the most widely used one for forecasting the transportation data, especially the short-term traffic flow forecasting [9]. It is a supervised learning algorithm that can be trained to learn a function between input features and the output, which is represented by the target to be predicted. The most widely used ANN-based models in short-term traffic flow forecasting are multilayer perception (MLP), backpropagation neural networks (BPNN), and radial basis function neural networks (RBFNN) [10]. The pros and cons of these models have been addressed in the literature [7, 11, 12].

Another development of artificial intelligence based-techniques is the combination of the ANN and other methods. Xia and Zhang [12] combined the strengths of discrete wavelet transform and ANN processing to achieve strong nonlinear approximation ability and then applied them to the short-term traffic volume forecasting. A traffic flow prediction model based on wavelet transform and fuzzy neural network was proposed in optimal control of the intelligent traffic system [13]. Instead of using backpropagation algorithm, the master-slave particle swarm optimization (PSO) was used to optimize the parameters of the prediction model. A deep learning-based traffic flow prediction method (neural networks with many layers) was proposed to represent traffic flow features for prediction [14]. The method can successfully discover the latent traffic flow feature representation, such as the nonlinear spatial and temporal correlations from the traffic data. A traffic flow prediction model based on the fuzzy *c*-mean clustering method (FCM) and the neural network was proposed [15]. The FCM can improve the accuracy and robustness of the model, while ANN can optimize the generalization ability of the model.

Although various methodologies have been applied to the traffic forecasting problem, the ultimate objective remains the same to obtain the forecasting result with high accuracy and robustness. Attention has also focused on improving the existing methodologies and models. ANNs have been found to be more effective than traditional methods in various application areas [16–18]. Other than that, the hybrid intelligent system, which is based on the combination of artificial neural networks and other intelligent techniques, has been proposed to take the full advantages of ANNs. Fuzzy systems are appropriate if sufficient expert knowledge about the process is available, while neural networks are useful if sufficient process data are available or measurable. The fuzzy neural network can effectively solve nonlinear problems [19] and is particularly useful in applications where classical approaches fail or too complicated to be used.

From the signal analysis point of view, the traffic flow can be considered as a linear combination of different traffic flow versus time frequencies. Every component of traffic flow corresponds to a range of frequencies. The wavelet transform is especially suitable for transient analysis because of its time-frequency characteristics with automatically adjusted time-window lengths. Recent studies have shown that the wavelet transform can be used as an effective tool for capturing important features and characteristics of the traffic flow.

On the other hand, new evolutionary algorithms, including biogeography-based optimization (BBO), inspired by the behavior of natural phenomena, were developed for solving optimization problems. Through the competitive results of benchmarking studies, these algorithms have been proven to be powerful and are considered to outperform the other well-known algorithms. The BBO, proposed by Simon [20], was inspired by the migration process of species. Since then, BBO has been used in solving various complicated problems and is considered to outperform other algorithms, such as genetic algorithms (GA), ant colony optimization algorithms (ACO) [21, 22].

Rather than choosing a single technique, it might be beneficial to take advantage of several individual techniques. The merit of BBO algorithm, the wavelet transform, the fuzzy system, and the success of ANNs have encouraged us to combine these techniques for forecasting traffic flow. The rest of the paper is organized as follows: Section 2 presents related techniques such as the fuzzy model, wavelet transform, BBO algorithm, and fuzzy wavelet neural network.  Section 3 is devoted to the proposed fuzzy neural network trained by BBO. A case application is presented in Section 4.  Section 5 reports results and discussion; finally, Section 6 gives the conclusion of the study.

## 2. Related Techniques

### 2.1. Fuzzy Model

The fuzzy logic model is an appropriate approach to model complex systems. It is a process of mapping from a given input to an output using the theory of fuzzy sets. Fuzzy logic systems have the ability to approximate any continuous function and deal with complex nonlinear systems with ill-defined conditions and uncertain factors [23]. Among the various fuzzy modeling techniques, Takagi–Sugeno–Kang (TSK) fuzzy model is one of the most popular ones because of its mathematical treatability. A TSK fuzzy model consisting of IF-THEN rules with fuzzy antecedents and a mathematical function at the consequent part of the form is as follows:

(1) $$\begin{array}{c} R^{i}:If\;x_{1}=A_{1}^{i}\text{and }x_{2}=A_{2}^{i}\text{ and}\ldots \text{and }x_{r}=A_{r}^{i},\\ \text{Then }y^{i}=a_{0}^{i}+a_{1}^{i}x_{1}+a_{2}^{i}x_{2}+\ldots +a_{r}^{i}x_{r},\text{ for i}=1,2,\ldots ,\text{K}, \end{array}$$

where *R* ^*i*^ represents the *i*th fuzzy inference rule, *K* is the number of rules, *x*_*j*_(*j*=1, 2, ..., *r*) is the *j*th input, *y* ^i^ is the output of the fuzzy rule, *A*_1_^*i*^,*A*_2_^*i*^,…, *A*_*r*_^*i*^ are fuzzy sets with membership functions *A*_*j*_^*i*^(*x*_*j*_), and *a*_*j*_^*i*^ s are real values. The output of the TSK fuzzy model is computed by

(2) $$\begin{array}{c} y=\frac{\sum_{i=1}^{K}\omega_{i}y_{i}}{\sum_{i=1}^{K}\omega_{i}}, \end{array}$$

where *ω*_*i*_ is the firing strength of rule *R* ^*i*^, which is calculated by

(3) $$\begin{array}{c} \omega_{i}=A_{1}^{i}\left(x_{1}\right)\times A_{2}^{i}\left(x_{2}\right)\times \cdots \times A_{r}^{i}\left(x_{r}\right). \end{array}$$

The fuzzy membership functions of *A*_*j*_^*i*^(*x*_*j*_) are Gaussian functions calculated by

(4) $$\begin{array}{c} \mu_{ij}\left(x_{j}\right)=\exp\left({\left(-\frac{x_{j}-c_{ij}}{\sigma_{ij}}\right)}^{2}\right), \end{array}$$

where *c*_*ij*_ is the center and *σ*_*ij*_ represents the standard deviation for fuzzy membership function associated with rule *i*.

It has been shown that the TSK fuzzy model can separate the input space into local fuzzy regions and then approximates a system in every region by a linear equation.

### 2.2. Wavelet Transform

The wavelet transform is a recently developed mathematical tool for signal analysis. It has been applied successfully in a wide range of time-series analysis, such as in astronomy, data compression, signal and image processing, earthquake prediction, and so on [24, 25]. The fundamental idea in wavelet analysis is to select a suitable wavelet (mother wavelet), and then perform an analysis using its translated and dilated versions. There are several kinds of wavelets that can be used as a mother wavelet, such as the Haar wavelet, Meyer wavelet, Coiflet wavelet, Daubechies wavelet, and Morlet wavelet. Each wavelet has specific characteristics.

Wavelets are as in the following form:

(5) $$\begin{array}{c} \psi_{a,b}={\left|a\right|}^{-1/2}\psi \left(\frac{x-b}{a}\right),\left(a,b\in R,a\neq 0\right). \end{array}$$

Wavelets are a family of functions derived from the function *ψ*(*x*) by the operation of dilation and translation. *ψ*(*x*) ∈ *L* ^2^(*R*) is a mother wavelet function that satisfies the following condition:

(6) $$\begin{array}{c} C_{\psi }=\int_{0}^{+\infty }\frac{{\left|\hat{\psi }\left(\omega \right)\right|}^{2}}{\omega }d\omega <+\infty , \end{array}$$

where $\hat{\psi }\left(\omega \right)$ is the Fourier transform of *ψ*(*x*).

The function *f*(*x*) can be represented by the following equation:

(7) $$\begin{array}{c} f\left(x\right)=\frac{1}{C_{\psi }}\iint Wf\left(a,b\right){\left|a\right|}^{-1/2}\psi \left(\frac{x-b}{a}\right)\frac{1}{a^{2}}dadb, \end{array}$$

where *Wf*(*a*, *b*)=|*a*|^−1/2^∫_−*∞*_^+*∞*^ *ψ*(*x* − *b*/*a*)*f*(*x*)*dx* is the continuous wavelet transform of *f*(*x*).

### 2.3. Fuzzy Wavelet Neural Network

**Figure 1:**
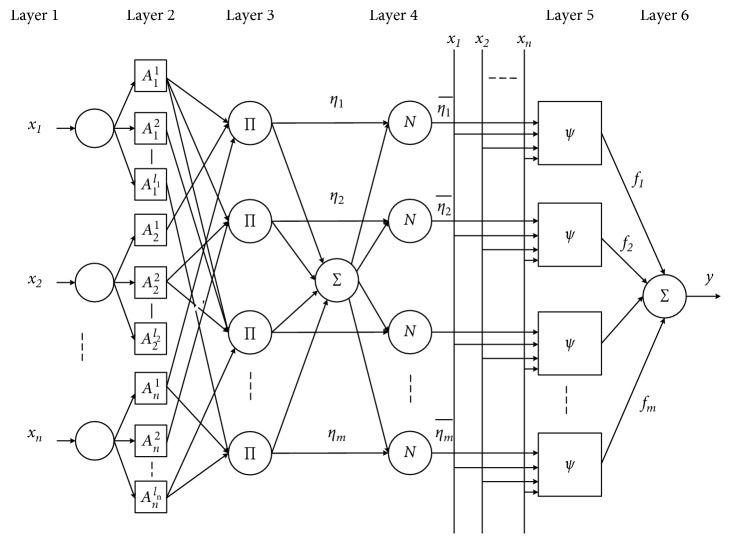
The structure of the FWNN.

The fuzzy wavelet neural network (FWNN) presented by Yilmaz and Oysal [26] and Linhares et al. [27] is utilized in this work to construct the traffic flow forecasting model. The six-layer structure of the FWNN is presented in Figure 1.

Layer 1: the input layer transfers the input signal *x*={*x*_1_, *x*_2_, ..., *x*_*n*_} to the second layer.

Layer 2: in the fuzzification layer, each neuron in this layer gets fuzzy membership functions in the IF part of the rules. The membership functions are parameterized according to the specific applications. The outputs of the fuzzification layer are the values of membership functions. The Gaussian membership function is the most used one:

(8) $$\begin{array}{c} A_{j}^{i_{j}}=\exp\left(-\frac{1}{2}{\left(\frac{x_{j}-\mu_{i_{j}}}{\sigma_{i_{j}}}\right)}^{2}\right),j=1,2,\ldots ,n\text{ and }i_{j}=1,2,\ldots ,l_{j}, \end{array}$$

Layer 3: this layer is the fuzzy rule layer (inference layer). Each neuron has a fuzzy rule. The output of the *l*th node *η*_*l*_ is obtained by aggregating *A*_*j*_^*i*^(*x*_*j*_) using the AND (t-norm):

(9) $$\begin{array}{c} \eta_{l}=\overset{n}{\underset{j=1}{\prod }}A_{j}^{i_{J}}\left(x_{j}\right), \end{array}$$

where *l*=*i*_1_, *i*_2_, ..., *i*_*n*_; *i*_1_=1, ..., *l*_1_, *i*_2_=1, ..., *l*_2_; ...; *i*_*n*_=1, ..., *l*_*n*_.

Each possible combination of input membership functions denotes a fuzzy rule. All fuzzy rules are summed up to the node placed between layers 3 and 4.

Layer 4: in the normalization layer, each neuron calculates the normalization value for the *l*th rule by using the following equation:

(10) $$\begin{array}{c} \bar{\eta_{l}}=\frac{\eta_{l}}{\sum_{i=1}^{m}\eta_{i}}\left(l=1,...,m\right). \end{array}$$

The output of this layer represents the contribution ratio of a rule to the final result.

Layer 5: the consequent layer calculates the weighted output value of a rule.

(11) $$\begin{array}{c} f_{l}=\bar{\eta_{l}}\Psi_{l},\left(l=1,...,m\right). \end{array}$$

In this study, the Mexican hat wavelet function is utilized in this layer as follows:

(12) $$\begin{array}{c} \Psi_{l}=\overset{n}{\underset{i=1}{\sum }}w_{il}\left(1-{\left(\frac{x_{i}-b_{il}}{c_{il}}\right)}^{2}\right)\exp\left(-\frac{1}{2}{\left(\frac{x_{i}-b_{il}}{c_{il}}\right)}^{2}\right). \end{array}$$

Layer 6: the output layer computes the overall output. All signals from the wavelet neurons are summed up.

(13) $$\begin{array}{c} y=\overset{m}{\underset{l=1}{\sum }}f_{l}. \end{array}$$

### 2.4. Biogeography-Based Optimization (BBO) Algorithm

Biogeography is the science which studies the geographical distribution of living species. BBO is a new inspired algorithm that is based on biogeography [20, 28]. Simon [20] developed the mathematical models of biogeography to solve optimization problems. In BBO, variables that determine the quality of habitat are called suitability index variables (SIVs), and each habitat is considered as an individual and has its habitat suitability index (HSI). SIVs are independent variables, and HSI depends on SIVs. Habitats with large HSI accommodate more species which are suitable for species living, and, conversely, a low-HSI habitat contains fewer species which are not suitable for species living. When the number of species in a habitat increases, there is a strong tendency for species to emigrate from crowded habitats to find new ones with better life-supporting conditions and lower population density than the old habitats. Habitats with low population density may accept a lot of new species from high-HIS habitats by providing adequate life-supporting characteristics. The objective function can be considered as HSI, and the evolutionary procedure of BBO is to acquire the solutions which maximize the HSI by using the immigration and emigration features of the habitats. The pseudocode of the BBO algorithm can be described in Algorithm 1.

 In BBO, the probability to choose the solution *H*_*i*_ as the immigrating habitat depends on its immigration rate *λ*_*i*_ and the probability to choose the solution *H*_*j*_ as the emigration habitat depends on its emigration rate *µ*_*j*_. Migration can be demonstrated as

(14) $$\begin{array}{c} H_{i}\left(\text{SIV}\right)⟵H_{j}\left(\text{SIV}\right). \end{array}$$

The immigration rate and emigration rate can be described as

(15) $$\begin{array}{c} \lambda_{i}=I\left(1-\frac{k_{i}}{n}\right),\\ \mu_{i}=E\left(\frac{k_{i}}{n}\right), \end{array}$$

where *I* and *E* are the maximum possible immigration rate and emigration rate, respectively. *k*_i_ represents the rank of habitat *i* after sorting all habitats according to their HSI and *n* is the number of solutions in the population. A better solution has higher emigration and lower immigration rates and vice versa.

**Figure 2:**
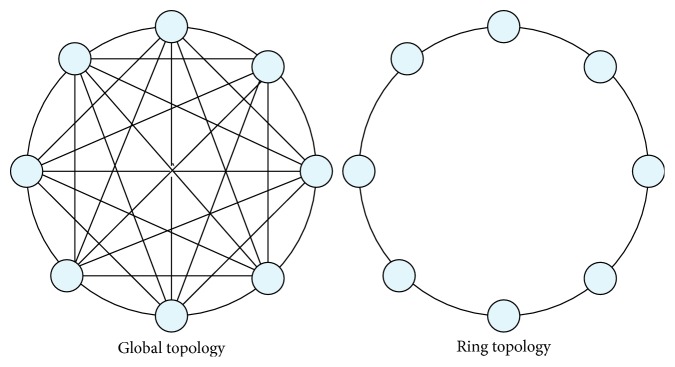
The global and local ring topologies.

The original BBO has several drawbacks including insufficient exploration capability and slow convergence speed. In order to improve the BBO, an attempt was made by combining the random ring topology and Powell's method [29]. The original BBO uses a global topology in which each pair of habitats can directly inform to the others. However, computing the distances between all pairs of habitats takes a high computational cost. A simplest form of local topology, called the ring topology, has been proposed to be used in BBO. In the ring topology, in order to reduce the computational cost and avoid premature convergence, each habitat is connected to only two other habitats, as shown in Figure 2 [30]. The pseudocode of the ring topology is presented in Algorithm 2.

Powell's method is utilized to effectively improve the solution precision. A perturbed best solution is used as the initial search point, and parameters *ε* and *δ* are the termination criteria and step size, respectively. The step size *δ*_*j*_ of the *j*th dimension can be derived from Equation 17:

(16) $$\begin{array}{c} \delta_{j}=\frac{0.0001.\sum_{i=1}^{m}\left(H_{i,j}-H_{best,j}\right)}{0.1ps}, \end{array}$$

where ps is the population size and 0.1 *ps* is the number of solutions selected for calculation, *H*_*i*_ presents the *i*th solution, and *H*_*best*_ is the best solution. The step size *δ*_*j*_ decreases when the number of iterations increases.

Moreover, the modified mutation proposed by Lohokare et al. [31] is also utilized to increase the population diversity. The detail of the improved BBO is represented in Algorithm 3.

## 3. The Proposed Fuzzy Wavelet Neural Network (FWNN) Trained by the Improved BBO

A fuzzy wavelet neural network (FWNN) with parameters trained by the improved BBO (hereinafter referred to as FWNN-iBBO-based model) was developed for forecasting the traffic flow. The parameters in the FWNN structure that need to be updated are as follows:

1. *μ*_*i*_*j*__ and *σ*_*i*_*j*__ (the center and the standard deviation for Gaussian fuzzy membership function associated with rule *i* in the layer 2, resp.)
2. The translation parameters *b*_*il*_ and dilation parameters *c*_*il*_ of wavelet functions
3. The weight parameters *w*_*il*_ in the consequent part the rules
4. The parameter vector Θ=(*μ*_*i*_*j*__, *σ*_*i*_*j*__, *b*_*il*_, *c*_*il*_, *w*_*il*_)

The FWNN parameters are updated according to the performance index of root-mean-squared error (RMSE) given in the following:

(17) $$\begin{array}{c} \mathrm{RMSE}\left(\Theta ,\tilde{x}\right)=\sqrt{\frac{1}{K}{\overset{K}{\underset{i=0}{\sum }}\left(y_{i}-{\hat{y}}_{i}\right)}^{2}}, \end{array}$$

where *y*_*i*_ is the actual (desired) value and ${\hat{y}}_{i}$ represents the forecasted value.

**Figure 3:**
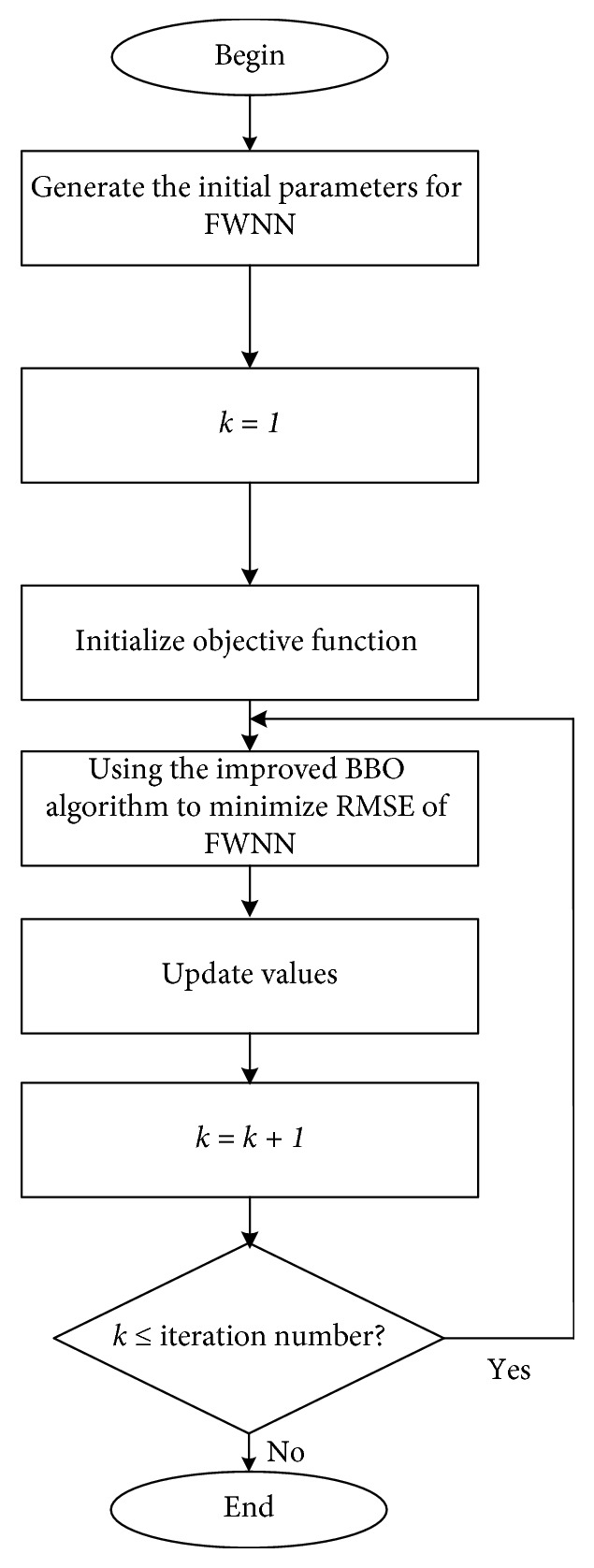
Training FWNN by iBBO.

In this study, the iBBO algorithm is utilized to train the forecasting model. The best parameters Θ=(*μ*_*i*_*j*__, *σ*_*i*_*j*__, *b*_*il*_, *c*_*il*_, *w*_*il*_) are selected based on the performance criteria. First, the whole data set is grouped into the training set and the testing set. After the training process (as shown in Figure 3), the trained FWNN based on the training set is applied to the testing set, and the performance criteria are recorded. The performance criteria are then applied to the trained model to estimate how well the trained model works. These criteria are used to compare forecasting values and actual values. They are as follows:

(1) Root-mean-squared error (RMSE): this index calculates the residual between the actual value and predicted value. A model has better performance if it has smaller RMSE. RMSE equal to zero means perfect fit.
  (18) $$\begin{array}{c} \mathrm{RMSE}=\sqrt{\frac{1}{n}{\overset{n}{\underset{i=0}{\sum }}\left(y_{i}-{\hat{y}}_{i}\right)}^{2}}, \end{array}$$
  where *y*_*i*_ is the actual value, ${\hat{y}}_{i}$ is the predicted value produced by the model, and *n* is the total number of observations.
(2) Mean absolute percentage error (MAPE): this index indicates an average of the absolute percentage errors. A model with the lower MAPE achieves the better performance:
  (19) $$\begin{array}{c} \mathrm{MAPE}=\frac{1}{n}\overset{n}{\underset{t=1}{\sum }}\left|\frac{y_{i}-{\hat{y}}_{i}}{y_{i}}\right|. \end{array}$$
(3) Correlation coefficient (*R*): this criterion indicates the strength of relationships between actual value and predicted value. The correlation coefficient has a range from 0 to 1, and a model with the higher *R* means it has better performance.
  (20) $$\begin{array}{c} R=\frac{\sum_{i=1}^{n}\left(y_{i}-\bar{y}\right)\left({\hat{y}}_{i}-\bar{\hat{y}}\right)}{\sqrt{\sum_{i=1}^{n}{\left(y_{i}-\bar{y}\right)}^{2}.\sum_{i=1}^{n}{\left({\hat{y}}_{i}-\bar{\hat{y}}\right)}^{2}}}, \end{array}$$
  where $\bar{y}=1/n\sum_{i=1}^{n}y_{t}$ and $\bar{\hat{y}}=1/n\sum_{i=1}^{n}{\hat{y}}_{t}$ are the average values of *y*_*i*_ and ${\hat{y}}_{i}$.

## 4. A Case Application

In this section, we use the traffic flow data from Ho Chi Minh City, Vietnam, to evaluate our developed model and compare the performance of our model with other models. Like other cities in Vietnam, Ho Chi Minh City is dealing with traffic problems characterized by mixed traffic flow including different categories of vehicles such as motorized and nonmotorized vehicles with the wide variation in sizes. All the vehicles including cars, buses, trucks, motorbikes, and bicycles are grouped into different categories, as shown in Table 1 [32]. Our study is aimed at forecasting traffic volume on the road from 621 T-junction to Thu Duc crossroad. The route is currently one of the busiest roads in Ho Chi Minh City (the largest city of Vietnam).

The total number of vehicles that pass over a given point during a given time interval is called volume. Traffic flow is the number of vehicles passing a reference point per unit of time, vehicles per hour. In the study, the numbers of vehicles crossing a fixed point of the road are counted. In mixed traffic, it is necessary to convert the whole traffic into one common standard or reference vehicle. For measuring the traffic volume, the car is selected as the reference vehicle. Area ratio is a criterion for finding the equivalent factor of the reference vehicle and the other vehicles.

(21) $$\begin{array}{c} \text{Area ratio}=\frac{A_{C}}{A_{Y}}, \end{array}$$

where *A*_*C*_ represents projected area of the reference vehicle (car) and *A*_*Y*_ denotes the projected area of “y” type vehicle. The area ratio for each vehicle is calculated and is presented in Table 2 [33].

**Figure 4:**
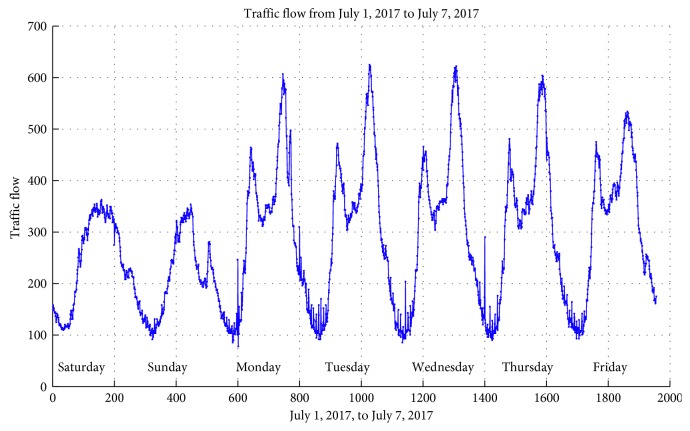
Traffic flow from July 1, 2017, to July 7, 2017.

A video recording of the forecasting section was done. The traffic flow datasets were then extracted from traffic cameras at a 15-minute interval. The data collection was conducted during the first six months of 2017 at an interval of 5 minutes. About 288 data samples were collected each day. The traffic flow in one week (from July 1 to July 7, 2017) on the monitoring site is shown in Figure 4. The traffic flow data show characteristic patterns tied to work-week activities. In workdays, the typical morning and evening peak hours are evident for urban routes. The evening peak has higher traffic volumes than the morning peak. Weekend days have lower-level peaks.

The traffic flow data were split into two parts: training data and testing data. Based on these data, several forecasting models are developed and evaluated.

**Figure 5:**
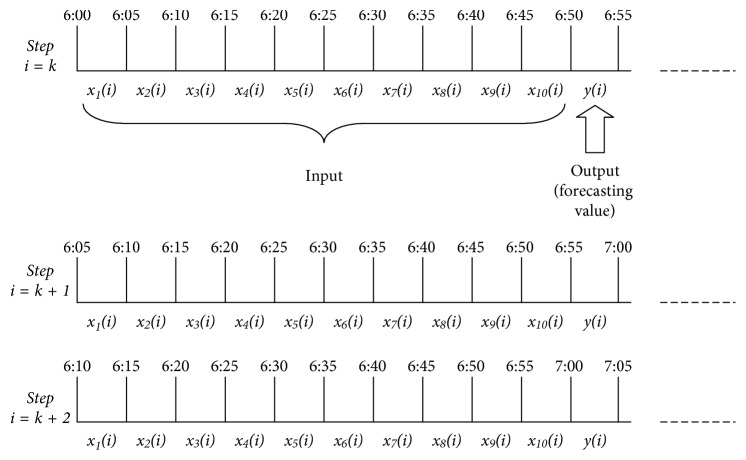
Illustration of 1-step traffic flow forecasting.

For each model, ten historical data points (a total of 50 minutes) are used as inputs and the output is the forecasting value for the traffic volume in the next five minutes. For instance, in Figure 5, if at Step i, the current time is 6 : 50, then the inputs for each forecasting model are the 5 minutes traffic flow data from 6 : 00 to 6 : 50, and the output is the traffic flow forecasting value from 6 : 50 to 6 : 55. At each succeeding step, a newly observed traffic flow value is added as the input and the oldest value is removed, such that the input dimension is constant.

## 5. Results and Discussion

**Figure 6:**
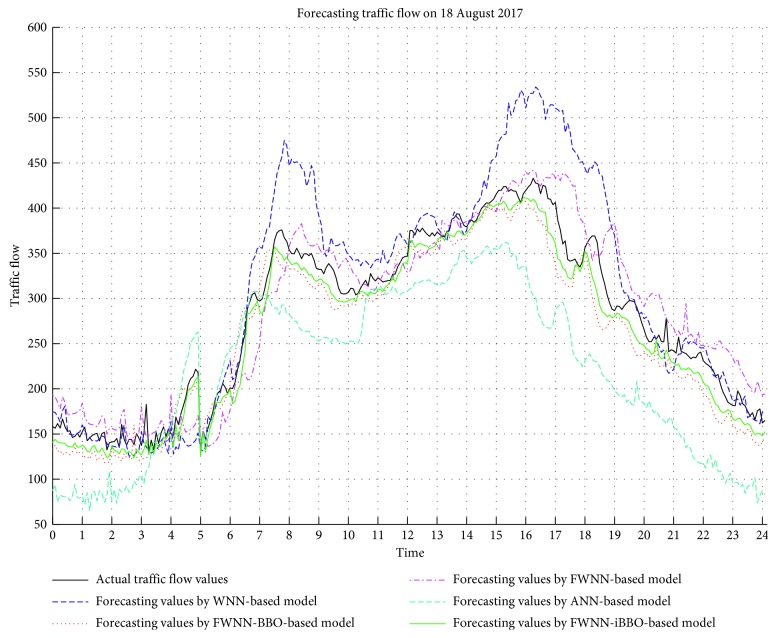
Forecasting traffic values by different forecasting models.

In this section, different forecasting models including FWNN-BBO-based model, ANN-based model, FWNN-based model, WNN-based model, and the proposed FWNN-iBBO forecasting model are developed and investigated. For each model, we conducted 10 independent tests, and each test produced a set of performance criteria values. The average performance criteria for each model were calculated and are presented in Table 3. The scattering diagrams and traffic flow graphics are also drawn in Figure 6. A fivefold cross-validation method was used to avoid an overfitting problem.

For ANN-based model, we adopt a feedforward network (FFN) with one hidden layer to forecast traffic volume. The optimum number of neurons in the hidden layer was determined by varying their numbers, starting with a minimum of one, and then increasing in steps by adding one neuron each time. Hence, various FFN architectures were tested to achieve the optimum number of hidden neurons. The best performing architectures for ANN were found to be 10-6-1. The activation function from input layer to hidden layer is sigmoid. With no loss of generality, a commonly used activation function, *f*(*n*) = 2/(1+*e* ^−2*n*^)–1, is utilized; while a linear function is used from the hidden layer to the output layer. The parameters for backpropagation were set as follows: the learning and momentum rates were 0.5 and 0.3, respectively. For the proposed FWNN-BBO forecasting model, the parameters for the BBO algorithm and iBBO algorithm were determined by trial and error. The parameters were set as follows: population size, *p*_*s*_=100; maximum immigration rate, *I* = 1; the maximum emigration rate, *E* = 1; mutation probability, *m*_max_ = 0.005; *ε* = 0.1; limit = 100; and *G* = 500.

**Figure 7:**
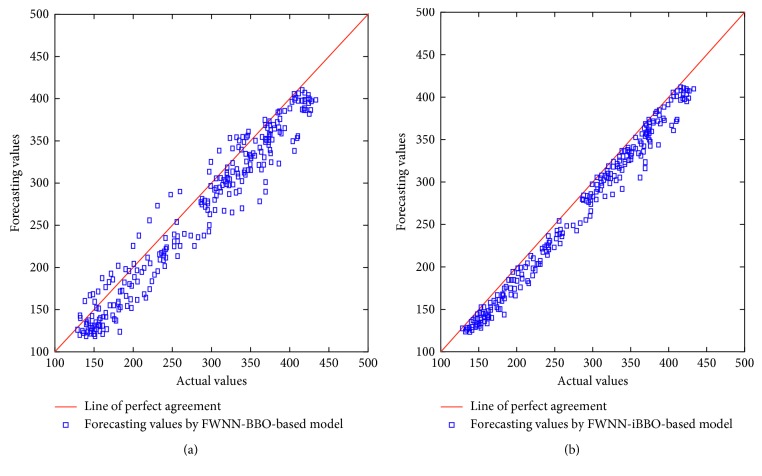
Forecasting values by FWNN-BBO-based model and FWNN-iBBO-based model.

The models were implemented in the MATLAB 2015a environment. The simulation results were then obtained and are presented in Figures 6 and 7 and Table 3. The time series of actual and forecasting values obtained by the WNN-based model, FWNN-BBO-based model, FWNN-iBBO-based model, FWNN-based model, and ANN-based model are compared in Figure 6. The nearly perfect agreement between the trends in the plots of the actual and forecasting values indicates that the FWNN-BBO-based model is the most suitable model.

The performance criteria RMSE, MAPE, and *R* obtained by FWNN-iBBO-based model were calculated as 20.4034, 0.0719, and 0.9846, respectively. Theoretically, a forecasting model is accepted as ideal when RMSE and MAPE are small, and *R* is close to 1. It is very clear from Table 3 that the FWNN-BBO-based model has a smaller RMSE and MAPE as well as a bigger *R* than those of the ANN-based model, FWNN-based model, and WNN-based model. These performance criteria indicate that the assessed results obtained by the FWNN-iBBO-based model are highly correlated and more precise.

The comparison between actual values and forecasting values obtained by FWNN-BBO-based model and FWNN-iBBO-based model are also shown in Figure 7. The figure presents the scatter diagrams that illustrate the degree of correlation between forecasting values and actual values. An identity line was drawn as a reference. In this figure, the identity line represents that the two sets of data are identical. The more the two datasets agree, the more the points tend to concentrate in the vicinity of the identity line. It may be observed that most forecasting values are very close to the actual values. This indicates a sound agreement between the forecasts by FWNN-BBO-based model and the actual values.

**Figure 8:**
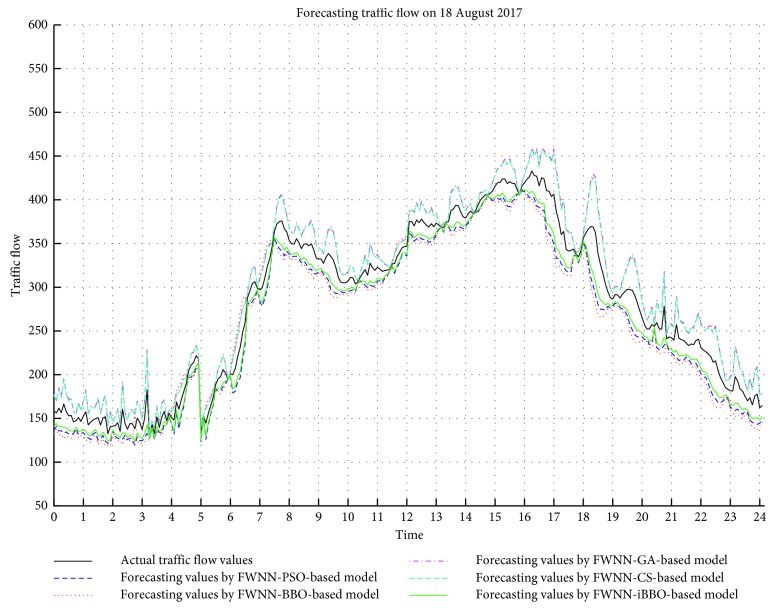
The forecasting values of FWNN trained by different optimization algorithms.

In order to evaluate the performance of the proposed approach, several popular and recent optimization algorithms including genetic algorithm (GA), particle swarm optimization (PSO), and cuckoo search (CS) algorithm were also applied to training FWNN (abbreviated as FWNN-GA, FWNN-PSO, and FWNN-CS). For each training algorithm, different sets of parameters were tried to obtain the best performance. For FWNN-GA, the population size was set at 30 and *p*_*c*_ and *p*_*m*_ were set at 0.6 and 0.4, respectively; and the number of iterations was set at 500. For FWNN-PSO, the number of initial population was set at 30 with *c*_*1*_ and *c*_*2*_ set to be 2, *w* decreased linearly from 0.9 to 0.4, and the initial velocities of particles were randomly generated from [0,1]. For FWNN-CS, the step size (*α*) was set at 0.01, the number of nests was set at 30, and the net discovery rate (*p*_*a*_) was set at 0.1. The results were recorded and depicted in Figure 8. As shown in the figure, the performances of FWNN-iBBO and the FWNN-BBO surpassed those of the FWNN-GA, FWNN-PSO, and FWNN-CS-based models. It can be concluded that the iBBO outperforms the GA, PSO, and CS algorithms in this study.

Based on the obtained results, it can be inferred that the proposed FWNN-iBBO-based model can be used to forecast the short-term traffic flow. The FWNN-iBBO-based model outperforms the FWNN-BBO-based model, ANN-based model, FWNN-based model, and WNN-based model, and the results show that its forecasting outcome is more accurate and reliable. Hence, the FWNN-iBBO-based model is acceptable and good enough to serve as a predictor of traffic flow.

## 6. Conclusions

In this study, different traffic flow forecasting models have developed and applied to forecast traffic flow on the road from 621 T-junction to Thu Duc crossroad which is characterized by mixed traffic flow. This study proposed a model based on fuzzy logic, wavelet transform, neural network, and the heuristic algorithm to forecast traffic flow. The results clearly demonstrated the superior forecasting performance of FWNN-iBBO model. It is concluded that FWNN can be utilized for short-term traffic flow prediction with mixed traffic conditions in Vietnam. The numerical experiments indicate the potential of the proposed method for large-scale network-wide traffic forecasting applications. As for the future research, it may be desirable to apply the proposed model to evaluate more traffic flow data from different locations. In addition, the proposed model can also consider additional factors, such as social events and weather to forecast the traffic flow.

## Figures and Tables

**Algorithm 1:**
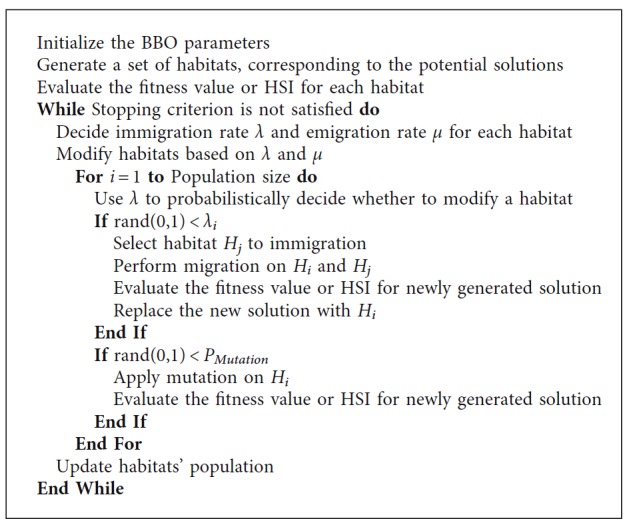
The BBO algorithm.

**Algorithm 2:**
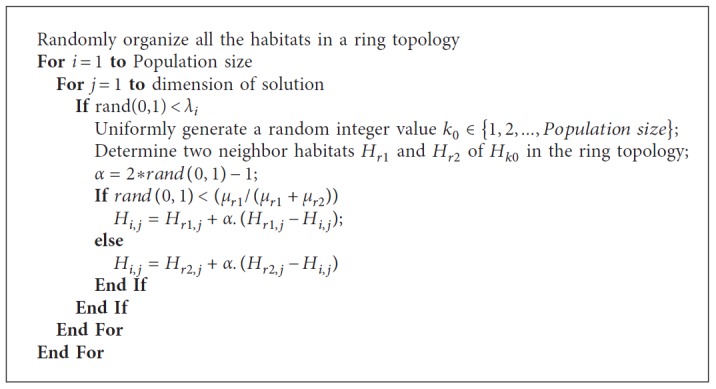
Pseudocode of the ring topology.

**Algorithm 3:**
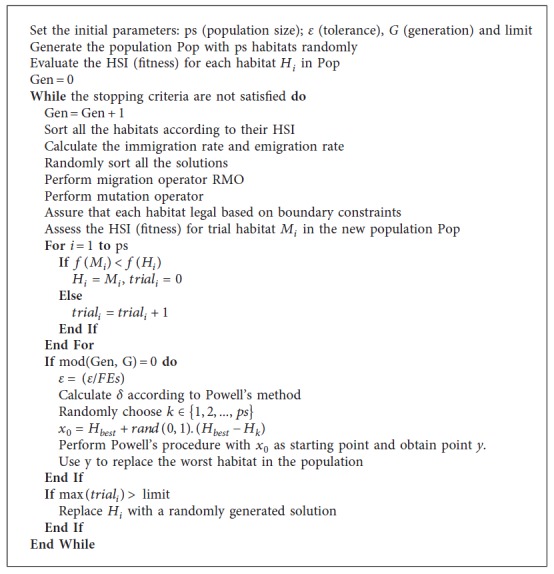
Pseudocode of the improved BBO.

**Table 1:** Vehicles and their dimensions.

Category	Vehicles included	Average dimension (m)		Projected rectangular on ground (m^2^)
		Length	Width	
Car	Car, jeep	3.72	1.44	5.39
Bus	Bus	10.1	2.43	24.74
Truck	Truck	7.5	2.35	17.62
Light commercial vehicle	Mini bus, van	6.1	2.10	12.81
Tractor	Tractor trailer	7.4	2.20	16.28
Two-wheeler	Scooter, motorbike	1.87	0.64	1.2

**Table 2:** Vehicles and their dimensions.

Category	Vehicles included	Projected rectangular on ground (m^2^)	Area ratio car (reference vehicle)
Car	Car, jeep	5.39	1.00
Bus	Bus	24.74	0.22
Truck	Truck	17.62	0.31
Light commercial vehicle	Mini bus, van	12.81	0.42
Tractor	Tractor trailer	16.28	0.33
Two-wheeler	Scooter, motorbike	1.2	4.49

**Table 3:** Performance statistics of different forecasting models.

	RMSE	MAPE	R
ANN-based model	74.0569	0.2529	0.8880
FWNN-based model	32.5282	0.1025	0.9451
FWNN-BBO-based model	27.4678	0.0924	0.9768
FWNN-iBBO-based model	20.4034	0.0719	0.9846
WNN-based model	52.1006	0.1171	0.9617

## Data Availability

The data used to support the findings of this study are available from the corresponding author upon request.
